# Pectin from Okra (*Abelmoschus esculentus* L.) Has Potential as a Drug Release Modifier in Matrix Tablets

**DOI:** 10.1155/2021/6672277

**Published:** 2021-01-15

**Authors:** Mariam El Boakye-Gyasi, Frederick William Akuffo Owusu, Philomena Entsie, Jacob Kwaku Agbenorhevi, Ben Kwaku Branoh Banful, Marcel Tunkumgnen Bayor

**Affiliations:** ^1^Department of Pharmaceutics, Faculty of Pharmacy and Pharmaceutical Sciences, Kwame Nkrumah University of Science and Technology, Kumasi, Ghana; ^2^Department of Herbal Medicine, Faculty of Pharmacy and Pharmaceutical Sciences, Kwame Nkrumah University of Science and Technology, Kumasi, Ghana; ^3^Department of Food Science and Technology, Kwame Nkrumah University of Science and Technology, Kumasi, Ghana; ^4^Department of Horticulture, Kwame Nkrumah University of Science and Technology, Kumasi, Ghana

## Abstract

Natural polymers have become attractive to pharmaceutical researchers and manufacturers as excipients because of the advantages they possess relative to their semisynthetic and synthetic counterparts. Although pectin from some natural sources has been investigated for use in the pharmaceutical industry as excipients, pectin from okra, which is readily available and used as food in many parts of the world, has not been extensively investigated as a potential control-releasing agent in tablets. This study thus seeks to determine the drug release modifying properties of okra pectin from 6 different genotypes of okra cultivated and available in Ghana. Pectin was extracted from different genotypes of okra, physicochemical properties were characterized, and control release matrix tablets of metformin (F1–F6) were formulated using the wet granulation method with the okra pectin as the drug release modifier, respectively. The drug content, *in vitro* drug release, and mathematical kinetic modeling of drug release from the matrix tablets were studied. Drug release profiles of formulated matrix tablets were compared to an existing (innovator) brand of metformin sustained-release tablet on the market using the similarity and difference factors, respectively. The extracted pectin had percentage yields ranging from 6 to 20% w/w with swelling indexes and water-holding capacities between 300–500% and 9-10 mL/g, respectively, and pH within 6.20–6.90. All the formulated batches passed the drug content test (90–105%) and produced the optimal release of metformin (>80%) after 24 hours. Different batches of formulated tablets exhibited different mechanisms of drug release with batches F1, F2, F5, and F6 being similar (ƒ_2_ values being >50 and ƒ_1_ values <15) to the innovator brand. Pectin from the 6 different genotypes of okra studied has the potential for use as drug release modifiers in pharmaceutical manufacturing of control release matrix tablets and production of more affordable medicines.

## 1. Introduction

Control release matrix tablets are designed to achieve a prolonged effect by continuously releasing the active pharmaceutical ingredient (API) over a long period of time. This prolonged release effect is provided by an integral excipient of the matrix tablet known as the release modifier [1–3]. Many synthetic polymers are available for use as release modifiers in the manufacture of controlled-release tablets. However, currently, pharmaceutical scientists are extensively investigating natural polymers as potential and reliable sources of release modifiers in controlled release tablets due to their myriad of advantages such as biodegradability, biocompatibility, low cost, low toxicity, and easy accessibility over synthetic and semisynthetic polymers [4–6]. Several studies have reported the potential of gum from different plants as promising release modifiers in the formulation of controlled release tablets [7, 8]. However, other plant hydrocolloids such as pectin may also possess remarkable potential as release modifiers in controlled release matrix tablets.

Pectin is a naturally occurring biopolymer found in plants and is finding increasing applications in the pharmaceutical and biotechnology industry. Pectin has been used successfully for many years in the food and beverage industry as thickening agent, gelling agent, and colloidal stabilizer. They are interesting candidates for pharmaceutical use, as carriers of a variety of drugs for controlled release applications [5, 6, 9, 10]. Commercially available pectin is mostly derived from citrus peel or apple pomace, both by-products from juice or cider manufacturing. Long period of maturity for the citrus plant and apple fruit may be a challenge. Various attempts have been made to extract pectin from plants with short maturity periods, which can produce a substantial yield of pectin. *Abelmoschus esculentus* (L.) Moench (Malvaceae), commonly called okra, has been a promising lead in this regard [11].

Okra (*Abelmoschus esculentus* L.) is a plant that is widely cultivated around the world [12]. About 50 species of okra have been identified in different parts of the world and have been investigated as excipients in pharmaceutical formulations [8, 10, 11]. Studies carried out by Emeje and Hussain et al. [13, 14] revealed the potential of okra gum as a binder in immediate release tablets. Similarly, Kumar et al. [15] reported of the potential disintegrating properties of okra gum while Ghori et al. [16] reported of the potential utilization of okra gum as a pharmaceutical excipient. However, studies on the potential of okra pectin and the effects of its genotypic variation as a pharmaceutical excipient are limited. Isolated okra pectin is rich in rhamnogalacturonan-I (RG-I) segments with varying composition of side chains and molecular weights. Pectin can exhibit marked differences in macromolecular characteristics, which affect their functional properties, depending on the genotype and source of the plant, stage of maturity, and extraction processes [5, 17].

In previous studies, pectin from orange peel, apple pomace, mango peel, and cocoa pod husk sources has been investigated as a carrier for targeted drug delivery and also as a control release agent for formulated metformin matrix tablets [18–22]. Studies have been published on okra pectin obtained from these six genotypes of okra in Ghana: Balabi, Agbagoma, Asha, Asontem, Sengavi, and Penkrumah [17]. However, the use of okra pectin from these 6 genotypes previously studied as drug release modifiers has not been reported.

Consequently, this study aims to investigate the drug release modification property of pectin extracted from six Ghanaian genotypes of okra from matrix tablets. Furthermore, using mathematical kinetic models, the mechanism of drug release is evaluated.

## 2. Materials and Methods

### 2.1. Chemicals and Reagents

Magnesium stearate, talc, and microcrystalline cellulose were purchased from Sigma-Aldrich. Deionized water was used throughout the experiments. Metformin powder was obtained from Ernest Chemists Limited, Ghana. All other reagents and chemicals used were of analytical grade.

### 2.2. Cultivation, Collection, and Authentication of Okra

The matured fruits of 6 genotypes of *Abelmoschus esculentus* L. (Malvaceae), namely, Balabi, Agbagoma, Asha, Asontem, Sengavi, and Penkrumah cultivated at the Department of Horticulture were harvested in April 2018. The collected genotypes were authenticated at the Department of Horticulture, KNUST, Kumasi, Ghana.

### 2.3. Extraction of Okra Pectin Samples

Okra pectin was extracted using a previously reported extraction protocol [17, 23]. The dried okra powder (20 g) was defatted with petroleum ether (1 g : 10 ml) by placing the okra ether mixture on a rotary shaker (120 rpm, 25°C) for 4 h. The defatted okra powder was subjected to aqueous extraction with 0.1 M phosphate buffer (1 g powder : 30 ml buffer solution), pH 6.0 at 80°C for 1 h. After extraction, the soluble polymer was separated from the insoluble residue by centrifugation (3000 rpm for 10 min at 25°C). The solubilized pectin in the supernatant was concentrated by evaporation at 80°C and then precipitated with 96% (v/v) aqueous ethanol at 40°C for 1 h (1 : 2). The extraction with ethanol was followed by washing using isopropanol and then freeze-dried. The percentage pectin yield was calculated based on the amount of dry okra powder sample used for the extraction process and the amount of dry okra pectin extract obtained [17, 24].

The extracted okra pectin samples from the six different genotypes were labelled as G1, G2, G3, G4, G5, and G6, respectively, corresponding to Balabi, Agbagoma, Asha, Asontem, Sengavi, and Penkrumah and stored in a desiccator for further experiments.

### 2.4. Physicochemical Characterization of Okra Pectin

#### 2.4.1. Determination of pH, Swelling Index, and Water-Holding Capacity

One gram of pectin from each genotype was weighed and added to 50 ml of distilled water and sonicated for 5 minutes. The mixture was topped up to 100 ml using distilled water and the pH was determined using a Hanna pH meter (HI 2215). The swelling index of pectin from each genotype was determined by weighing one gram into a 10 ml measuring cylinder and noting the initial volume (*V*_*i*_). Distilled water was added to the 10 ml mark. The measuring cylinder was stoppered, mixed lightly, and allowed to stand for 24 hours. The volume occupied by the pectin after 24 hours was also noted (*V*_*f*_):

(1) $$\begin{array}{c} \text{swelling index}=\frac{\left(V_{f}-V_{i}\right)}{V_{i}}\times 100. \end{array}$$

The contents of the measuring cylinders from the swelling index procedure were used in determining the water-holding capacity [9, 25].

#### 2.4.2. Determination of Solubility Properties

The solubility properties of pectin from the various genotypes were determined at 25°C in hot water, cold water, petroleum ether, diethyl ether, methanol, and ethanol. One gram of pectin per genotype was added to 50 ml of each solvent and allowed to stand overnight. Twenty-five milliliters of supernatant was placed in preweighed Petri dishes and evaporated to dryness over a water bath and dried to a constant weight. The residue mass obtained was expressed as the percentage solubility of the pectin in the respective solvents [9, 26].

#### 2.4.3. Moisture Content Determination

Four grams of pectin from each genotype was weighed accurately into porcelain crucibles, which had previously been dried to a constant weight. The pectin was placed in a hot air oven and maintained at a temperature of 105°C. After 5 hours, the pectin was removed and cooled after which they were placed in a desiccator for 30 minutes. The weights of both crucibles and pectins were recorded [9, 25].

#### 2.4.4. Determination of Flow Properties of Okra Pectin

Twenty grams of powdered pectin from each genotype was weighed and emptied through a funnel into a measuring cylinder (100 ml). The initial volume (*V*_*o*_) was noted. The measuring cylinder was tapped on a wooden bench until the powders consolidated to a constant volume (*V*_*f*_). The Hausner ratio and Carr's index were subsequently calculated. The angle of repose was also determined using the fixed height method [27].

### 2.5. Fourier Transform Infrared Spectroscopy

The metformin and okra pectin samples were scanned individually over a wavenumber range of 4000 to 400 cm^−1^ using Bruker alpha II Fourier transform infrared spectroscopy. The samples of each tablet formulation containing both metformin and okra pectin and other excipients were also scanned to assess possible interactions that would show as a change in spectra.

### 2.6. Preparation of Tablets

The wet granulation method was employed in formulating matrix tablets (batches F1, F2, F3, F4, F5, and F6) using extracted okra pectin as drug release modifier. Each tablet contained 500 mg of metformin hydrochloride with the composition shown in Table 1 (eighty tablets were prepared for each batch). The concentration of okra pectin used was based on the outcome of a series of trial formulations. Microcrystalline cellulose was used as diluent while magnesium stearate and talc were used as glidant and lubricant, respectively. All the ingredients except the magnesium stearate and talc were weighed and mixed together. Wet mass was formed by adding water as the granulating fluid while mixing thoroughly. The wet mass was screened through a 2.36 mm mesh sieve to form the granules. The wet granules were dried for 1 hour at 60°C and screened through a 1.18 mm mesh sieve and then stored in airtight containers. The granules were compressed into tablets using a single punch tableting machine. Magnesium stearate and talc were added prior to compression. Six different batches of tablets (F1, F2, F3, F4, F5, and F6) using 20% w/w okra pectin from (G1 to G6) were formulated. All tablets were stored for further analysis.

### 2.7. Flow Properties of Granules

The flow and compressibility properties of the granules were evaluated prior to tablet compression; the angle of repose was determined using the fixed height method; the bulk and tapped densities were used for the determination of compressibility index as well as the Hausner ratio [27].

### 2.8. Evaluation of Formulated Matrix Tablets

#### 2.8.1. Uniformity of Weight Test

Twenty tablets were randomly chosen from each batch and weighed cumulatively and independently using a digital analytical balance (Mettler, B634930296). The average weight of the weighed tablets was calculated and the weight of each tablet was deducted from the average weight to determine the percentage deviation of the individual tablet from the average weight [26, 28].

#### 2.8.2. Crushing and Tensile Strength of Tablets

The dimensions of 20 randomly selected tablets from each batch were determined with the digital Vernier caliper (Mitutoyo, CD-8-CSX). The hardness of six randomly selected tablets from each batch was determined individually by diametrically compressing the tablets using a Veego hardness tester (HT-1). The tensile strength (Ts) of the randomly selected tablets was subsequently determined [15, 28, 29].

(2) $$\begin{array}{c} \text{tensile strength}=\frac{2}{3}\left[\frac{10F}{\pi D^{2}\left[2.84\left(t/D\right)-0.126\left(t/W\right)+3.15\left(W/D\right)+0.01\right]}\right], \end{array}$$

where *F* is the crushing strength, *D* is the length of the axis, *t* is the overall thickness, and *W* is the tablet height.

### 2.9. Friability Test

The friability test was performed using the C-FT-20 friability test apparatus. Ten tablets were randomly sampled from each batch, weighed, and placed in the friabilator, which was then regulated at 25 revolutions per minute. The tablets were subjected to one hundred (100) revolutions. Tablets were subsequently dedusted and reweighed to calculate the percentage weight loss [26, 28]. The percentage friability was calculated as follows:

(3) $$\begin{array}{c} \%\text{friability}=\frac{\left(W_{1}-W_{2}\right)}{W_{1}}\times 100, \end{array}$$

where *W*_1_ is the original weight before the friability test and *W*_2_ is the final weight after the friability test.

### 2.10. Drug Content of Formulated Matrix Tablets

Ten randomly selected tablets from each batch were used for the test. The tablets were powdered, and an equivalent weight of 500 mg metformin was transferred into a 100 ml volumetric flask and shaken with 70 ml of distilled water for 15 minutes and further diluted with water to 100 ml and filtered. A quantity (1 ml) of filtered solution was diluted to 100 ml with water, and the amount of drug in each sample was analyzed using a validated HPLC method [30, 31] with a Perkin Elmer Flexar HPLC with UV spectrophotometer at 232 nm. Each measurement was carried out in triplicate and the mean was taken. Drug concentration was subsequently calculated using a standard calibration curve [28, 30].

### 2.11. *In Vitro* Drug Release from Formulated Matrix Tablets

*In vitro* drug dissolution studies were conducted using the USP-XXII dissolution apparatus-2 (Electrolab, Mumbai, India) at a rotational speed of 100 revolutions per minute at 37 ± 0.5°C. The dissolution media were 900 mL of 0.1 M HCl for the first 2 h followed by phosphate buffer solutions (pH 6.8). Aliquots of dissolution medium (10 ml) were withdrawn manually at specific time intervals 0, 2, 4, 6, 8, 10, 16, 18, 21, and 24 hours, respectively. Sink conditions were maintained. The amount of drug in each sample was analyzed using a validated HPLC method with a UV spectrophotometer at 232 nm [30, 31]. Drug dissolved at specified time periods was plotted as cumulative percent release versus time (h). The dissolution test was performed in triplicate.

### 2.12. Release Kinetics and Mechanism of *In Vitro* Drug Release from Formulated Matrix Tablets

Mathematical modeling of the release kinetics of metformin from the matrix tablets was assessed by employing zero-order, first-order, Higuchi, Hixson–Crowell, and Korsmeyer–Peppas models, respectively [21].

Mathematical equations of the kinetic models employed are as follows:

(4) $$\begin{array}{c} \text{zero}-\text{order model}:\text{Qt}=kt+C\text{,}\\ \text{first}-\text{order model}:\text{In}\left(1-\text{Qt}\right)=-kt+C,\\ \text{Higuchi model}:\text{Qt}=kt1/2+C,\\ \text{Hixson}–\text{Crowell model}:{\text{Qo}}^{1/3}-{\text{Qt}}^{1/3}=K_{\text{HC}}t,\\ \text{Korsmeyer}–\text{Peppas}:\text{Qt}/\text{Q}\infty =ktn\text{,} \end{array}$$

where Qo was the initial amount of drug in the tablets, Qt was the amount of drug released during time *t*; *k* was a kinetic constant measuring the release rate; Qt/Q∞ was the fractional drug release at time *t*; n was the diffusional exponent which characterizes the release mechanism, *C* was a constant, and *K*_HC_ was release rate constant for Hixson–Crowell model [32].

### 2.13. Statistical Analysis

The results are presented as the mean ± standard deviation. Data were analyzed using GraphPad Prism version 6.00 for Windows (GraphPad Software, San Diego, California, USA). At 95% confidence interval, *p* ≤ 0.05 was considered significantly different. Dissolution profiles were compared using similarity factor (*f*_2_) and difference factor (*f*_1_).

## 3. Results and Discussion

### 3.1. Physicochemical Properties of Extracted Okra Pectin

**Figure 1:**
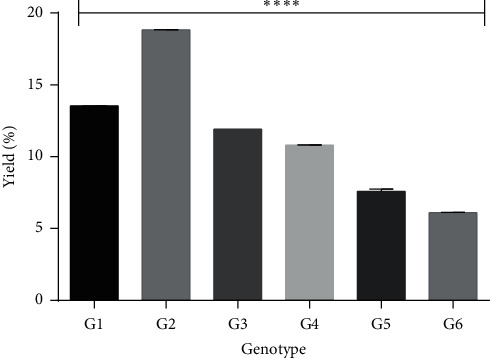
Yield of pectin obtained from the various genotypes. One-way ANOVA on ^*∗∗∗∗*^*P* < 0.0001.

The yield of pectin is affected by the plant used, the stage of maturity of the plant, the geographical location of the plant, and the extraction processes involved. It has also been reported that variations in the genotype of plant species can affect pectin yield [33, 34]. There were significant differences (*P* < 0.0001) in the yield of pectin obtained from the various genotypes (Figure 1). Since the same extraction protocol was used, the differences in the pectin yield indicate variations in the content of pectin from the various genotypes and confirm the fact that genotypic variations in a plant can affect pectin yield.

Solubility is a physical property, which can easily depict the potential of a polymer to be utilized as a pharmaceutical excipient (suspending, emulsifying, or control-releasing agent). Polymers with complete solubility in aqueous solvents may not be suitable as suspending or control release agents in pharmaceutical formulations. Solubility profile is also a means of characterizing a polymer and preventing adulteration. Pectin extracted from all the genotypes was sparingly soluble in aqueous solvents (Table 2). This indicates that they can become hydrated without dissolving completely in aqueous media and can provide control-releasing properties.

**Figure 2:**
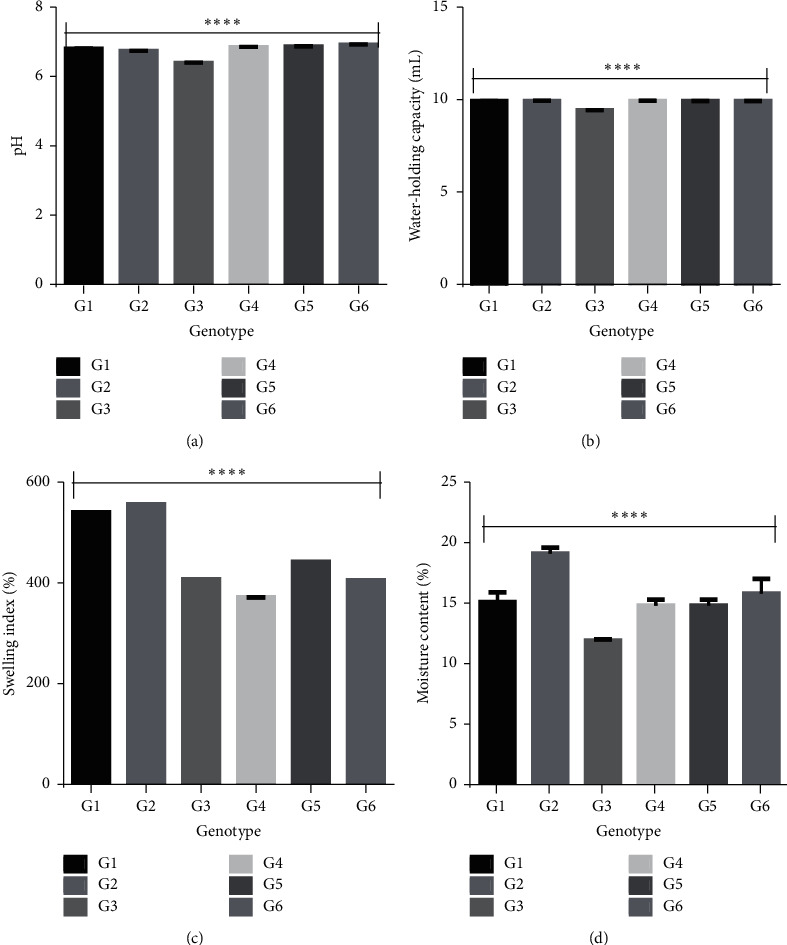
Physicochemical properties of extracted okra pectin with one-way ANOVA on the (a) pH, (b) water-holding capacities, (c) swelling indices, and (d) the moisture content of pectin obtained from the 6 genotypes of okra (^*∗∗∗*^*P* < 0.0001).

The physicochemical property of an excipient affects the overall quality of a finished pharmaceutical product. There were significant differences (*P* < 0.0001) in the pH, water-holding capacity, swelling index, and moisture contents of extracted pectin from the different genotypes. These significant differences may result in pectin from one genotype being more suitable for a specific function in a pharmaceutical dosage form as compared to the other genotypes (Figure 2).

**Figure 3:**
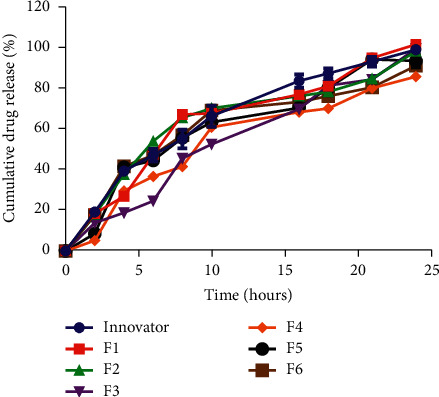
*In vitro* drug release from formulated matrix tablets and innovator brand of metformin SR tablets^∗^.

The water-holding capacity, swelling index, and moisture content of pectin play very key roles in predicting the characteristics of the pectin. Hydrophilic polymers used as control-releasing agents act by hydration and swelling to produce a gel, which gradually erodes to produce the control-releasing property. Polymers with good swelling indexes exhibit good control-releasing properties. This implies that a hydrophilic matrix with a faster polymer hydration capability will swell up quickly and exhibit good control-releasing and drug-releasing properties. This was observed in the drug release profiles as G4 had the least swelling index and also produced the formulation (F4) with the least drug release from the second hour until the twenty-fourth hour (Figure 3).

### 3.2. Interaction between Metformin and Okra Pectin

**Figure 4:**
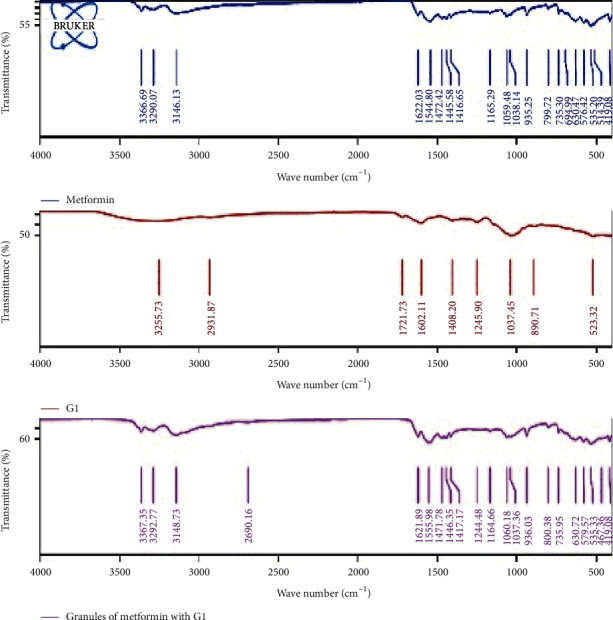
FT-IR spectra of metformin, G1, and granules of metformin with G1 over the frequency range 4000–400 cm^−1^.

The FTIR spectra of metformin only, pectin from the different genotypes of okra, and granules made from metformin and the different pectin samples, respectively, showed no disappearance of any characteristic functional peaks. Thus, there was no interaction between metformin and any of the pectin samples or excipients in the various tablet formulations (Figure 4).

### 3.3. Properties of Formulated Tablets

The granules compressed into the various batches of tablets exhibited good flow properties (Table 3). The formulated matrix tablets showed uniformity of weight conforming to pharmacopoeial standards. Tablet hardness, friability, and tensile strength were within acceptable limits and the assay of the tablets showed that they all contained the required amount of metformin according to the British Pharmacopoeia stated limits (Table 4).

### 3.4. Drug Release Modifying Properties of Okra Pectin

Comparative *in vitro* drug release patterns of formulated matrix tablets and innovator brand of metformin matrix tablets in acidic and buffer phase showed that all the formulated batches were able to provide a controlled drug release over the stipulated twenty-four-hour period (Figure 3). Thus, pectin from the 6 different genotypes has potential as control-releasing agents, respectively. To assure similarity in product performance between an innovator and newly formulated products, *in vitro* dissolution profiles can be compared using the difference (*f*_1_) and similarity (*f*_2_) factors [35, 36]. Two dissolution profiles are found to be similar, if *f*_1_ value is between 0 and 15 and *f*_2_ value is between 50 and 100. The dissolution profile of batches F1, F2, F5, and F6 was similar to that of the innovator with F2 being the most similar while the dissolution profile of batches F3 and F4 was not similar to that of the innovator (Table 5) [37].

### 3.5. Mathematical Kinetic Modeling of Metformin Release from Matrix Tablets

Generally, polymeric matrix swelling, material degradation, and solute diffusion are implicated as the main driving forces for transport of solute from a polymeric matrix system containing an active pharmaceutical ingredient (API) [32, 38, 39]. Thus, variations in the rate of polymer swelling and degradation as a result of differences in physicochemical properties will affect the rate of solute diffusion and invariably produce different release kinetics. The extracted pectin from the various genotypes showed different kinetics of drug release: Fickian diffusion (F1, F5, and F6), super case II transport (F2), zero order (F3), or first order (F4), respectively, for the different samples (Table 6) which confirms that the significant differences in their physicochemical properties (Figure 2) affect the rate at which they release the active pharmaceutical ingredient (API). An ideal matrix system will provide a constant release of the drug (zero order) and this was provided by F3 (Table 6). While there are many methods that scientists may use to achieve zero-order release, most of them are complicated, costly, time-consuming, and difficult to produce [40]. Thus, the ability of F3 to produce a zero order will be very beneficial for pharmaceutical manufactures of controlled release matrix tablets. A large number of release modifiers provide a first-order release kinetics (where time has an effect on surface area and drug diffusional path length), which was also depicted by batch F4 (Table 6). However, due to the intricate and complex processes involved in drug dissolution from matrix systems, majority of these systems produce Fickian diffusion and specialized transport of the active pharmaceutical ingredient (API) which were also depicted by F1, F5, F6, and F2, respectively [32, 41].

## 4. Conclusion

Pectin from the 6 different genotypes of okra exhibited varying degrees of control release behaviour and the mathematical modeling of drug release by pectin from the different genotypes of okra showed different mechanisms of drug release. Thus, pectin from the 6 different genotypes of okra studied may be used as drug release control agents in pharmaceutical manufacturing as well as the food and other industries.

## Figures and Tables

**Table 1:** Composition of matrix tablet formulations.

Ingredients	Master quantities (mg)
Metformin	500.0
Okra pectin (G1–G6)	190.0
Microcrystalline cellulose	260.0
Talc	9.0
Magnesium stearate	4.5

**Table 2:** Solubility properties of Okra pectin in various solvents.

	Hot water	Cold water	Petroleum ether	Diethyl ether	Methanol	Ethanol
G1	+++	+++	+	+	++	++
G2	+++	+++	+	−	−	+
G3	+++	+++	+	++	+	+
G4	+++	+++	+	+	++	+
G5	+++	+++	−	+	++	++
G6	+++	+++	−	−	++	+

Note: +++: sparingly soluble; ++: slightly soluble; +: very slightly soluble; −: practically insoluble.

**Table 3:** Flow properties of granules formulated.

Sample	Hausner ratio	Carr's index	Angle of repose (^o^)
G1	1.069 ± 0.001	4.76 ± 0.021	27.11 ± 0.098
G2	1.111 ± 0.006	10.31 ± 0.080	30.13 ± 0.036
G3	1.131 ± 0.004	11.55 ± 0.049	33.22 ± 0.124
G4	1.120 ± 0.011	10.73 ± 0.054	31.09 ± 0.059
G5	1.095 ± 0.001	8.68 ± 0.001	28.77 ± 0.069
G6	1.130 ± 0.008	11.33 ± 0.046	32.98 ± 0.001

**Table 4:** Properties of formulated tablets.

	Tablet weight uniformity (mg)	Tablet hardness (*N*)	Tablet friability % weight loss	Tablet tensile strength (MPa)	Drug content of formulated matrix tablets (%)
F1	0.966 ± 0.021	78.42 ± 1.486	0.02	1.2668 ± 0.041	98.45
F2	0.966 ± 0.004	74.39 ± 2.333	0.03	1.2043 ± 0.022	98.96
F3	0.966 ± 0.003	78.20 ± 0.118	0.02	1.2513 ± 0.019	97.08
F4	0.965 ± 0.001	76.20 ± 1.123	0.04	1.2218 ± 0.026	98.98
F5	0.966 ± 0.013	77.20 ± 0.045	0.05	1.2336 ± 0.045	101.14
F6	0.965 ± 0.011	78.20 ± 0.067	0.04	1.2835 ± 0.032	99.63

**Table 5:** Difference factor (*f*_1_) and similarity factor (*f*_2_) between innovator brand and formulated tablets.

Formulation	Difference factor (*f*_1_)	Similarity factor (*f*_2_)	Comment
F1	1.41	59.68	Similar
F2	1.22	60	Similar
F3	16.89	45.17	Dissimilar
F4	19	45.48	Dissimilar
F5	6.53	59.83	Similar
F6	6.17	57.82	Similar

**Table 6:** Drug release kinetics of metformin in the various matrix tablet formulations.

Formulations	Zero order (*r*^2^)	First order (*r*^2^)	Higuchi (*r*^2^)	Hixson–Crowell (*r*^2^)	Korsmeyer–Peppas (*r*^2^)
F1	0.9045	0.8883	0.9627	0.7833	0.7437
F2	0.8417	0.7558	0.9571	0.8923	0.9609
F3	0.9786	0.6405	0.9450	0.7980	0.7790
F4	0.9119	0.9772	0.9579	0.9699	0.8888
F5	0.9009	0.8983	0.9604	0.9498	0.6889
F6	0.8645	0.9316	0.9647	0.9364	0.9300

## Data Availability

The data used to support the findings of this study are available from the corresponding author upon request.
